# Identifying a Highly-Aggressive DCIS Subgroup by Studying Intra-Individual DCIS Heterogeneity among Invasive Breast Cancer Patients

**DOI:** 10.1371/journal.pone.0100488

**Published:** 2014-06-30

**Authors:** Dana Pape-Zambito, Zhengyu Jiang, Hong Wu, Karthik Devarajan, Carolyn M. Slater, Kathy Q. Cai, Arthur Patchefsky, Mary B. Daly, Xiaowei Chen

**Affiliations:** 1 Cancer Epigenetics Program, Fox Chase Cancer Center, Philadelphia, Pennsylvania, United States of America; 2 Department of Pathology, Fox Chase Cancer Center, Philadelphia, Pennsylvania, United States of America; 3 Department of Biostatistics and Bioinformatics, Fox Chase Cancer Center, Philadelphia, Pennsylvania, United States of America; 4 Department of Clinical Genetics, Fox Chase Cancer Center, Philadelphia, Pennsylvania, United States of America; University of New Mexico, United States of America

## Abstract

The heterogeneity among multiple ductal carcinoma *in situ* (DCIS) lesions within the same patient also diagnosed with invasive ductal carcinoma (IDC) has not been well evaluated, leaving research implications of intra-individual DCIS heterogeneity yet to be explored. In this study formalin-fixed paraffin embedded sections from 36 patients concurrently diagnosed with DCIS and IDC were evaluated by immunohistochemistry. Ten DCIS lesions from each patient were then randomly selected and scored. Our results showed that expression of PR, HER2, Ki-67, and p16 varied significantly within DCIS lesions from a single patient (*P*<0.05 for PR; *P*<1×10^−8^ for HER2, Ki-67 and p16). In addition, seventy-two percent of the individuals had heterogeneous expression of at least 2/6 markers. Importantly, by evaluating the expression of promising DCIS risk biomarkers (Ki-67, p53 and p16) among different DCIS subgroups classified by comparing DCIS molecular subtypes with those of adjacent normal terminal duct lobular units (TDLU) and IDC, our results suggest the existence of a highly-aggressive DCIS subgroup, which had the same molecular subtype as the adjacent IDC but not the same subtype as the adjacent normal TDLU. By using a systematic approach, our results clearly demonstrate that intra-individual heterogeneity in DCIS is very common in patients concurrently diagnosed with IDC. Our novel findings of a DCIS subpopulation with aggressive characteristics will provide a new paradigm for mechanistic studies of breast tumor progression and also have broad implications for prevention research as heterogeneous pre-invasive lesions are present in many other cancer types.

## Introduction

In 2014, it is estimated that about 62,570 new cases of breast carcinoma *in situ* will be diagnosed in the US, with the majority being classified as ductal carcinoma *in situ* (DCIS), which represents about one-fifth of the number of mammographically detected breast cancers in the US [1], [2]. DCIS is morphologically defined as a neoplastic proliferation of mammary epithelial cells that are confined to the ductal-lobular structures of the breast without invasion through the basement membrane. As a result, DCIS is generally not immediately life threatening. However, it is estimated that 14–53% of women diagnosed with DCIS subsequently develop invasive ductal carcinoma (IDC) if the DCIS is left untreated or inadequately treated [3]–[5]. Current standard treatment options for DCIS include surgery (lumpectomy or mastectomy) plus radiation (for lumpectomy) and an optional tamoxifen treatment (for patients with estrogen receptor positive [ER+] DCIS) [6]. Since there is no accurate risk assessment currently available to determine which patients with DCIS are at the greatest risk of developing invasive carcinoma in their lifetime, DCIS poses a primary challenge for physicians to make the best and safest treatment decision for patients with DCIS; whether they need surgery, radiation, and/or adjuvant hormone therapy. Uncertainties about the clinical behavior of DCIS often lead to unnecessarily aggressive treatment for DCIS patients with lesions that are unlikely to progress to invasive ductal carcinoma (IDC). This results in net harm to these breast cancer patients.

Although some clinical characteristics suggest the prediction of high-risk DCIS, such as architectural pattern, cell necrosis, and nuclear grade [7], [8], accurate assessment of the risk of DCIS progression is currently not possible. Molecular mechanisms that drive *in situ* malignant epithelial cells to progress to invasive cells are still not fully understood. More than 30 years ago, Wellings and Jensen et al. [9], [10] proposed a breast tumorigenesis model where IDC development follows a linear pattern from premalignant hyperplastic breast lesions with/without atypia, to carcinoma *in situ* (e.g., DCIS) and ultimately invasive breast cancer. Yet the behavior of DCIS is inconsistent and tremendous variability exists in the propensity of DCIS to progress to IDC [11]. Conventional comparisons between “pure” DCIS (i.e. without IDC for at least five years after initial DCIS diagnosis) and DCIS with IDC often require a large sample size to overcome the heterogeneity of DCIS among individuals [12]. Studies using a small sample size of either “pure” DCIS or DCIS with IDC are often statistically insignificant. To date, only a few peer-reviewed publications have reported DCIS risk assessment based on “pure” DCIS, but the results from these studies have been inconsistent [13]–[15]. The intra-individual heterogeneity in DCIS lesions is expected to explain discordant results between a core biopsy specimen and surgical or resection specimens, as reported previously [16], [17].

The various outcomes of DCIS makes it clinically relevant to establish an accurate risk assessment system for DCIS, but it also presents a major technical challenge. Tremendous progress on computational modeling of DCIS has recently been made which allows one to predict the size of a tumor using immunohistological and calcification characteristics of DCIS from a biopsy sample [18], [19]. This information can help the surgeon when removing lesions with DCIS, but still does not provide information on whether or not the DCIS will transition to IDC. In the study presented here, we tested the hypothesis that classification of heterogeneous DCIS based on adjacent IDC and normal terminal duct lobular unit (TDLU) in patients concurrently diagnosed with IDC and DCIS helps to identify DCIS subgroups which have different degrees of “aggressiveness”. By evaluating the expression of clinical biomarkers in a subset of IDC-DCIS cases, we first quantified how common heterogeneity is among multiple DCIS lesions from an individual patient concurrently diagnosed with IDC. We then compared the expression of promising DCIS risk biomarkers (Ki-67, p53 and p16) among the different DCIS subgroups, classified based on adjacent TDLU and IDC, to examine whether an “aggressive” DCIS subpopulation existed. Results from our proposed studies are expected to establish new approaches for DCIS biomarker discovery by taking advantage of the heterogeneous nature of premalignant lesions within the same breast cancer patient.

## Materials and Methods

### Subjects

Under a protocol approved by the Institutional of Review Board (IRB) at Fox Chase Cancer Center (FCCC), archived breast cancer cases accrued between 2007–2011 were selected from the FCCC tumor registry database. The database was queried for cases of IDC where DCIS was also reported in the surgical pathology reports. Some of the cases had an extensive intraductal component (EIC). Only anonymized and de-identified materials were used in our study, and no patient identifiers were used when analyzing data or reporting study results. Hematoxylin and eosin (H&E) stained slides from excisional biopsy or mastectomy blocks from these IDC-DCIS cases were evaluated by a board certified pathologist to confirm the presence of multiple DCIS lesions (>10) in the cases selected for final study. A DCIS lesion is defined as a single duct filled with ductal carcinoma cells, which are still bound by a myoepithelial cell layer. Cases with few histologically confirmed DCIS lesions were eliminated from the study. Paraffin blocks containing multiple DCIS lesions from each case were requested and 20 unstained serial sections were cut from each block. The results of the biomarker studies were correlated with the morphology of the specimen using an additional section stained with H&E.

### DCIS Lesion Selection

To randomly select DCIS lesions in each case, H&E sections were first scanned using an Aperio ScanScope CS 5 slide scanner (Aperio, Vista, CA) with a 20× microscope objective. Scanned images were then viewed with Aperio's image viewer software (ImageScope, version 11.1.2.760), which allows the user to review the H&E sections at 1, 2, 4, 5, 10, and 20× magnifications. Using the scanned images, initially all DCIS lesions from a whole tissue section were identified and a board certified pathologist confirmed the lesions were DCIS. Ten individual DCIS lesions were then randomly selected throughout the entire tissue section for subsequent marker scoring. In instances where a differential diagnosis of IDC was considered, staining with p63 antibody was performed to ensure that the ductal carcinoma cells were still bound by a myoepithelial cell layer.

### Antibodies for Immunohistochemistry (IHC)

Breast tissue sections were stained with standard clinical receptor markers, ER, PR, and HER2, as well as three promising DCIS risk biomarkers (Ki-67, p16 and p53) [14]. Antibodies used for IHC were as follows: p53 (1∶500; clone D0-7), p16 (1∶50; clone EP1551Y), Progesterone Receptor (PR) (1∶400; clone Y85), and Estrogen Receptor (ER) (1∶100; clone SP1) were purchased from Abcam (Cambridge, MA). HER2 (1∶1,500, rabbit polyclonal) and Ki-67 (1∶100, clone Mib-1) were purchased from Dako (Carpinteria, CA). p63 (1∶2,000; clone 4A4) was purchased from Santa Cruz (Santa Cruz, CA). Cleaved Caspase-3 (Asp175) (1∶200) was purchased from Cell Signaling Technology (Danvers, MA). Goat anti-mouse and anti-rabbit secondary antibody systems were purchased from Dako (Cat. K4007 and K4011, respectively). Standard protocols for IHC were followed as described previously [20]. Mayer's Hematoxylin (BioGenex Cat. HK100-9k) was used to counterstain slides and visualize nuclei. Slides were coverslipped using Permount.

### Marker Scoring

All slides were viewed with a Nikon Eclipse 50i microscope, and photomicrographs were taken with an attached Nikon DS-Fi1 camera. Scores for ER, PR, and HER2 were based on scoring guidelines used by FCCC pathologists from 2007–2011. H scores for ERs and PRs in DCIS lesions were generated by multiplying the staining intensity of nuclei (0, 1, 2, 3) by the percentage of positive cells (0–100%). H-scores that were below 50 were considered negative for ER and PR, whereas H-scores above 50 were considered positive for ER and PR. Membranous expression of HER2 in DCIS lesions was scored on a scale of 0–3: no membrane staining (score of 0), light and incomplete membrane staining (score of 1), light to moderate membrane staining with clearly defined intercellular borders or strong complete membrane staining in <30% of cells (score of 2), strong and complete membrane staining in >30% of the cells (score of 3). HER2 staining was classified as low or negative if the score was 0 or 1, and HER2 was classified as high or positive if the score was 2 or 3. Ki-67 and p53 scoring was based on the percentage of cells with strongly stained nuclei: 0–10% positive cells (scored as 1), 11–50% positive cells (scored as 2), and >50% positive cells (scored as 3). For p53 staining only lesions containing cells with very strongly stained nuclei, which was indicative of mutant p53, were assigned a numeric score; all other lesions were considered as expressing wild type p53 (score 0). If strong nuclear staining (indicating mutant p53) was observed, lesions were given a numeric score as follows: 1–10% positive cells (scored as 1), 11–75% positive cells (scored as 2), and >75% positive cells (scored as 3). H-scores for p16 were determined by multiplying the intensity of stained cells (0, 1, 2, 3) by the percentage of positive cells. To further aid the classification, H-scores were grouped: 0–100 were redefined as 1, 101–200 as 2, and scores from 201–300 as 3. H-scores for Cleaved Caspase-3 were also determined by multiplying the intensity of cytoplasmic and perinuclear staining (0, 1, 2) by the percentage of positive cells. Staining of Cleaved Caspase-3 was then classified in two categories: negative-to-low (if H-score <2) or medium-to-high (if H-score ≥2). Staining intensity for ER, PR, HER2, Ki-67, p53, p16, p63 and Cleaved Caspase-3 are demonstrated in **Figure S1 in File S1**. All lesions were independently scored by two trained observers. Discrepancies between reviewers' scores were resolved by re-visiting slides and/or scoring from a third independent reviewer.

### Intra-Individual DCIS Heterogeneity

In this study, we sought to determine whether or not staining patterns of biomarkers vary in DCIS lesions within a single patient. Thus, a precise definition of variability for each marker was essential. Cases in which the scores of ≥20% of the lesions scored within the same individual differed from those of the majority of the lesions in that case were classified as having variable expression. All markers (ER, PR, HER2, Ki-67, p16 and p53) were scored as either variable or non-variable for each of the examined cases (n = 36). Secondly, to determine if patients have DCIS lesions that exhibit heterogeneity among multiple IHC markers, we set three different cutoff values to distinguish different levels of heterogeneity. When 0–1 markers in an individual patient were variably expressed, the case was classified as homogeneous. In contrast, when patients had 2/6 markers with variable expression, these patients were classified as having a moderate-degree of heterogeneous expression. A higher cutoff value of ≥3/6 markers with variable expression was used to denote patients with a high-degree of heterogeneous expression. For example if a patient had variable expression in PR, p16 and Ki-67, this patient would be considered to have a high-degree of DCIS heterogeneity.

### DCIS Classification

Based on ER, PR and HER2 scoring results from clinical pathology reports and our IHC experiments, DCIS lesions and adjacent TDLU and invasive components were classified as four different molecular subtypes; luminal A-like (ER+ and/or PR+, HER2−), luminal B-like (ER+ and/or PR+, HER2+), HER2+-like (ER−, PR−, HER2+), and basal-like or triple-negative (ER−, PR−, HER2−). Only immunostains for HER-2 were considered for molecular subtype analysis as FISH data was not available for the DCIS lesions. Additionally, heterogeneous DCIS lesions from each case were further categorized into two subgroups based on the molecular subtypes comparison with adjacent IDC: (i) DCIS Subgroup I, in which the DCIS molecular subtypes differed from the adjacent IDC, and (ii) DCIS Subgroup II, which shared the same molecular subtypes with the adjacent IDC. Lastly, Subgroup II DCIS lesions were further divided into Subgroups II*a* and II*b* based on the molecular subtypes of adjacent TDLU: Subgroup II*a* had the same molecular subtypes as both adjacent IDC and TDLU, whereas Subgroup II*b* had the same molecular subtypes as the adjacent IDC but not the same molecular subtypes as the adjacent TDLU. We then compared the expression of three promising DCIS biomarkers, Ki-67, p16, and p53, among these DCIS subgroups.

### Statistical Analysis

In order to determine if the proportion of cases exhibiting variable expression for a single marker was higher than our hypothesized value of 10%, a one-sided exact binomial test was done at the 5% significance level. In other words, we tested the hypothesis that the percentage of heterogeneity in DCIS lesions is significantly higher than 10%. The 10% base line is the cut-off to define homogeneity among DCIS lesions, i.e., variability of 10% or less could be caused by artifacts of IHC staining, but variability of >10% could represent true biological differences. This 10% value is more conservative than a previous study of heterogeneity, where the baseline for homogeneity was defined as 5% of cells having different nuclear grades [21]. Independent statistical analyses were performed for ER, PR, HER2, Ki-67, p16, and p53. To examine the correlations of ER scores (positive and negative) and those of other markers in the DCIS lesions, Fisher's exact test was used. A *p*-value of 0.05 or less was considered significant. We also applied logistic regression models involving multiple IHC markers (e.g. Ki67, p53 and p16) to identify the models(s) that best predicted the DCIS sub-group. Single and multiple marker models were tested. Lesions within a single patient were treated as being independent and each model was adjusted for the effect of multiple lesions within the patient. For each model, we computed the receiver operating characteristic (ROC) curve. The area under the ROC curve (AUC) quantifies the ability of the test to correctly classify DCIS sub-groups. We evaluated the predictive performance of each model using the AUC and its 95% confidence interval.

## Results

### Clinical Sample Information

One-hundred fifteen patients with concurrent DCIS and IDC were identified in the Fox Chase Cancer Center tumor registry database between 2007 and 2011, of which 46 patients had extensive histologically confirmed DCIS. Blocks were cut and IHC was performed on all cases. After evaluating the quality of the IHC staining as well as the presence of p63 positive myoepithelial cells surrounding DCIS lesions, 36 patients were used in this study. All women had an invasive cancer (1 well, 13 moderately, and 22 poorly differentiated), and tumor size averaged 3.3 cm. Twenty-one patients had nodal involvement of at least 1 lymph node. The characteristics of the invasive cancers in the study population are summarized in **Table 1**. There were no significant differences in age, tumor size, grade, lymph node involvement, and HER2 status between ER+ and ER- tumors (relevant to hormonal therapy). PR status was significantly associated with ER status (*P*<0.00001), as expected.

**Table 1:** Clinical sample information of the invasive cancer components classified by ER status in patients with IDC and DCIS (n = 36).

Clinical Characterizations	ER+ (n = 25)	ER- (n = 11)	*P*-Value^b^
***Age at diagnosis (yr); mean***	48	51	
***Tumor size (cm); mean***	3.2 (range 0.8–6.0)	3.3 (range 1.2–9.0)	
***Tumor grade***			
*Well differentiated*	1 (4.0%)	0 (0%)	
*Moderately differentiated* a	11 (44%)	2 (18.2%)	
*Poorly differentiated*	13 (52%)	9 (81.8%)	*0.15*
***Lymph node involvement***			
*No* a	12 (48%)	3 (27.3%)	
*Yes*	13 (52%)	8 (72.7%)	*0.30*
**Clinical Immunohistochemistry**			
***PR***			
*Positive* a	22 (92%)	1 (9.1%)	
*Negative*	3 (8.0%)	10 (90.9%)	***0.00001***
***HER2***			
*Positive* a	5 (20%)	3 (27.3%)	
*Negative*	20 (80%)	8 (72.7%)	*0.68*

a Reference category.

b Fisher's exact test.

### Intra- Individual DCIS Heterogeneity is Common at Both the Single-Marker and the Molecular Subtype Levels

**Figure 1:**
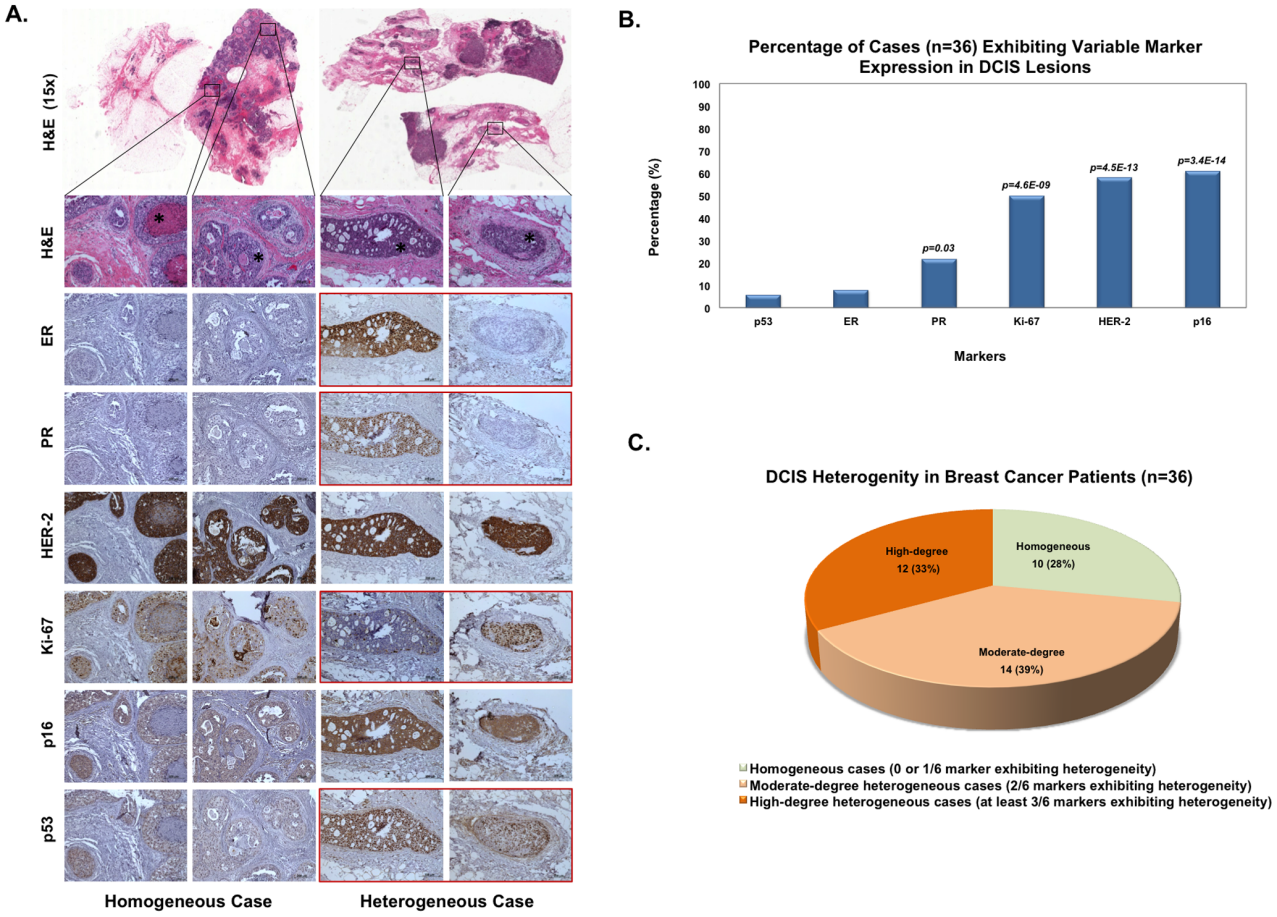
Evaluation of intra-individual DCIS heterogeneity among patients (n = 36) concurrently diagnosed with IDC and DCIS. (**A**) Examples of intra-individual homogeneity and heterogeneity in IHC marker expression of DCIS lesions. In an example of a homogeneous case (left), expression of all markers is similar across DCIS lesions. All markers within this case were classified as having no variable expression; therefore, this case is not considered to have intra-individual heterogeneity in the DCIS lesions. The example of a heterogeneous case on the right panels has variable expression of ER, PR, Ki-67, and p53 (highlighted in red; not all lesions shown in figure). Because more than three markers exhibited variable expression, this case has a high-degree of heterogeneity within the DCIS lesions. All but the top panels were taken at 100× magnification. (**B**) Percentage of cases exhibiting variable expression of IHC markers. For each IHC marker (i.e., p53, ER, PR, Ki-67, HER2, or p16) in an individual case to be considered as having variable expression, at least 20% of the DCIS lesions were required to have different expression levels compared to the majority of lesions. *P*-values represent how significant the differences are relative to a hypothesized baseline value of 10% variability in staining. (**C**) Intra-individual heterogeneity in DCIS. Cases were considered as having a moderate- or high-degree of heterogeneity if at least 2/6 or 3/6 markers, respectively were classified as having variable marker expression.

Intra-individual heterogeneity was defined by comparing the number of markers with variable expression to the total number of markers evaluated. **Figure 1A** illustrates examples of homogeneity and heterogeneity in IHC marker expression of DCIS lesions. Fewer than 10% of the cases exhibited a variable expression pattern for ER and p53, therefore these markers tended to be similar among all DCIS lesions within a single patient (**Figure 1B**). Conversely, expression of PR, HER2, Ki-67, and p16 were variable in 22, 58, 50 and 61% of cases, respectively, which significantly differed from our hypothesized baseline value of 10% of the cases demonstrating variable expressions of markers (*P*<0.05 for PR; *P*<10^−8^ for HER2, Ki-67 and p16).

**Figure 2:**
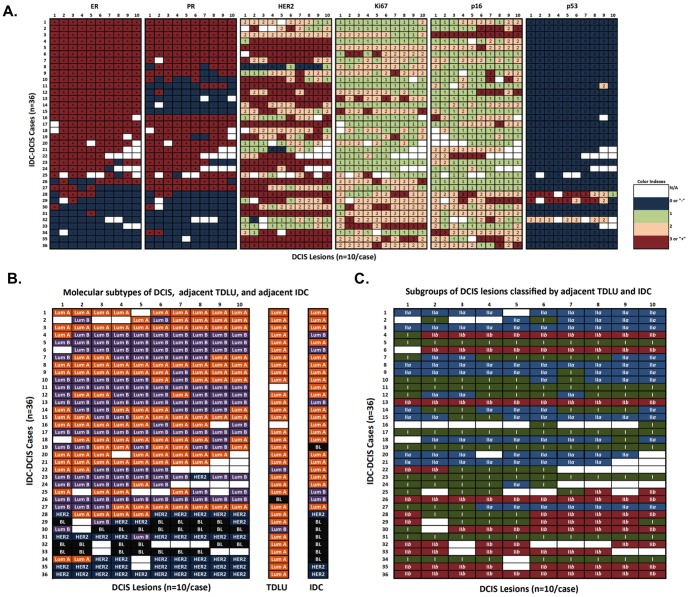
IHC marker scores and DCIS classification among individual DCIS lesions from patients with IDC and DCIS (n = 36). (**A**) Heat map of IHC markers for DCIS lesions. This heat map illustrates marker scoring for each individual DCIS lesion across six IHC markers. The individual lesions are aligned in columns and the unique patients are aligned in rows. (**B**) Molecular subtypes of DCIS lesions, adjacent TDLU, and IDC for each case. Subtypes including luminal A-like (ER+ and/or PR+, HER2−), luminal B-like (ER+ and/or PR+, HER2+), HER2+-like (ER−, PR−, HER2+), and basal-like/triple negative (ER−, PR−, HER2−) are classified according to hormone receptor status. *(Lum: luminal; BL: basal-like; TDLU: terminal duct lobular units; blank: data is not available)* (**C**). By comparing DCIS lesions with the molecular subtypes of adjacent IDC and the hormone receptor status in adjacent TDLU, DCIS lesions from each IDC-DCIS case were first classified into two subgroups based on the molecular subtypes of adjacent IDC: DCIS Subgroup I, which presented different molecular subtypes from the adjacent IDC; DCIS Subgroup II, which presented the same molecular subtypes as adjacent IDC. Then the Subgroup II DCIS lesions were further divided into Subgroup II*a* (with the same molecular subtypes as both adjacent IDC and TDLU) and Subgroup II*b* (with the same molecular subtypes as the adjacent IDC but not the same subtypes as the adjacent TDLU).

The relative degree of intra-individual heterogeneity among DCIS lesions was determined for each patient (n = 36). A patient was considered to have intra-individual DCIS heterogeneity when at least 2 of the 6 markers tested presented with variable expression. If none or only one marker exhibited variable expression, the case was classified as homogeneous. As shown in **Figure 1C**, 72% of the cases (26 out of 36) displayed some degree of intra-individual heterogeneity in DCIS lesions, strikingly higher than our hypothesized value of 10% of cases demonstrating heterogeneity (*P*<10^−18^). In addition, DCIS lesions that were positive for ER staining were also more likely to be PR+ (*P* = 3.3×10^−22^), whereas ER- DCIS lesions had moderate or high Ki-67 staining (*P* = 2.3×10^−10^ and *P* = 4.3×10^−8^, respectively) (**Figure 2A** and **Table S1 in File S1**). Lesions with p53 mutations, as indicated by the dark brown staining (**Figure S1 in File S1**), were also more likely to be ER- (*P* = 3.2×10^−8^). ER status was not associated with HER2 IHC scores in DCIS lesions, which is consistent with the clinical data from the invasive ductal carcinoma components presented in **Table 1**. As previous studies have shown that there is a possible correlation between Ki-67 and Cleaved Caspase-3 staining in breast cancer [22], [23], we also evaluated the IHC staining patterns between Ki-67 and Cleaved Caspase-3 in the DCIS lesions. As shown in Table S2 in File S2, there is a positive correlation between Ki67 and Cleaved Caspase-3 staining (*P*<5×10^−7^). The average percentage of Cleaved Caspase-3 positive cells in individual DCIS lesions from our patient sample set (n = 36) is about 2%, which is consistent with the findings from a previous report [24]. The percentage of Cleaved Caspase-3 positive cells is much lower than those of Ki-67 positive cells (∼30%) in DCIS, suggesting that cell proliferation dominates over apoptosis in DCIS.

Previous studies indicated that DCIS displays four molecular subtypes similar to IDC [25], [26], including luminal A, luminal B, HER2+, and basal-like, which are based upon ER, PR and HER2 status [27], [28]. To examine DCIS heterogeneity among various breast cancer molecular subtypes, we classified the lesions by molecular subtype, i.e. luminal A-like (ER+ and/or PR+, HER2−), luminal B-like (ER+ and/or PR+, HER2+), HER2+-like (ER−, PR−, HER2+), and basal-like/triple negative (ER−, PR−, HER2−), for each individual DCIS lesion based upon our ER, PR and HER2 staining results. Using the same definition of variability as stated previously (≥20% of the lesions scored within an individual case differed from those of the majority of lesions in that case), we determined that 38.9% cases (14 out 36) had DCIS lesions with variable molecular subtypes (**Figure 2B**). Furthermore, we compared the molecular subtypes of the IDC component, obtained from clinical pathological reports, with our classification of the DCIS components. Not surprisingly, greater than 50% of heterogeneous cases had different molecular subtypes when comparing adjacent IDC and DCIS components, but only 25% of homogeneous cases displayed different molecular subtypes when comparing adjacent IDC and DCIS.

### Clinical Implications of DCIS heterogeneity

In addition, we also sought to determine whether the intra-individual DCIS heterogeneity correlates with prognosis of patients with IDC and DCIS (n = 36). The difference in overall or recurrence-free survival between the homogeneous group and the heterogeneous group was not significant (**Figure S2 in File S2**). However, when examining whether the degree of intra-individual heterogeneity of DCIS lesions was associated with clinical characteristics (i.e., grade, tumor size, node involvement as well as clinical results of receptor status) in the patients concurrently diagnosed with IDC and DCIS, we found that a high-degree of heterogeneity but not a moderate degree of heterogeneity in DCIS lesions was nearly associated with positive nodal involvement in comparison to the homogeneous group (*P* = 0.07, borderline, **Table 2**). This result indicates that those patients with a high level of marker heterogeneity may be more likely to have more aggressive or metastatic cancers that are difficult to treat.

**Table 2:** Associations between clinical characteristics and intra-individual DCIS heterogeneity in patients with IDC and DCIS (n = 36).

Intra-individual Heterogeneity	Grade		Tumor Size		Node Involvement	
	W/M^b^	Poor (*P* c)	<3 cm	≥3 cm (*P*)	Yes	No (*P*)
Homogeneous^a^	3	7	7	3	4	6
Moderate	8	6 (*0.24*)	7	7 (*0.42*)	7	7 (*0.70*)
High	3	9 (*1.00*)	5	6 (*0.39*)	10	2 (*0.07*)

a Reference category.

b W  =  well differentiated and M  =  moderately differentiated.

c P-value (Fisher's exact test).

### The Distribution of DCIS Markers Differed among the Subgroups of Heterogeneous DCIS

The expression patterns of the non-receptor markers (Ki-67, p16 and p53) were examined in each lesion and by DCIS molecular subtypes. As shown in **Table 3**, we found that HER2+ and basal-like subtype DCIS lesions have higher Ki-67 and p53 scores than DCIS lesions with the luminal A subtype (*P*<10^−3^ and *P*<10^−2^, respectively). These findings are consistent with the previously published data for IDC [29], [30]. As DCIS represents an intermediate step in the transition of normal ducts to IDC during breast tumor development, DCIS lesions are expected to carry some of the same molecular signatures of normal ducts and/or invasive components. Therefore, we believe that comparing the molecular subtypes of adjacent TDLU and IDC with DCIS lesions will provide a novel approach to evaluate heterogeneity in DCIS, and could ultimately lead to the discovery of distinct subgroups of DCIS lesions. Consequently, we determined the molecular subtype of adjacent TDLU using ER, PR, and HER2 staining results using the categories described above. As reported previously [31], [32], TDLU of adjacent morphologically normal breast tissue was generally positive for ER and/or PR in a dispersed pattern, while being negative or displaying low levels of expression for HER2. As shown in **Figure 2B**, 91% of the TDLU (31 out of 34 cases) had a “Luminal A” subtype. Furthermore, we used the results from the clinical pathology reports to determine the molecular subtype of the IDC component. A comparison was then done to determine similarities and differences in molecular subtypes of the TDLU, DCIS, and IDC components. DCIS lesions with different molecular subtypes from patients concurrently diagnosed with IDC and DCIS were first categorized into two subgroups: Subgroup I DCIS (different molecular subtypes than the adjacent IDC) and Subgroup II DCIS (sharing the same molecular subtypes as the adjacent IDC). Based on the molecular subtypes of adjacent normal TDLU, Subgroup II DCIS lesions were further divided into Subgroups, II*a* (sharing the same molecular subtypes as the adjacent TDLU) and II*b* (with different molecular subtypes than the adjacent TDLU) (**Figure 2C**).

**Table 3:** Association between IHC marker scores and molecular subtypes among individual DCIS lesions from patients with IDC and DCIS (n = 36).

Molecular Subtypes	Ki-67			p16			p53	
	Low^a^	Moderate (*P* b)	High (*P*)	High^a^	Moderate (*P*)	Low (*P*)	Wild-type^a^	Mutant (*P*)
Luminal A^a^	68	51	4	20	32	67	120	3
Luminal B	74	44 (*0.43*)	11 (*0.17*)	12	39 (*0.06*)	75 (*0.17*)	129	2 (*0.67*)
HER2+	8	31 (***5.0×10^−8^***)	6 (***7.7×10^−8^***)	7	32 (*0.06*)	6 (*0.59*)	37	8 (***3.6×10^−8^***)
Basal-like	0	18 (***8.1×10^−8^***)	8 (***1.3×10^−8^***)	3	9 (*0.52*)	13 (*1.00*)	15	9 (***2.6×10^−8^***)

a Reference category.

b P-value (Fisher's exact test).

We then tested if any breast cancer markers, Ki-67, p16 or p53, have significant distribution differences among DCIS subgroups. As shown in **Table 4**, there were no significant distribution differences for Ki-67 index and p16 IHC staining between DCIS type I and type II*a* lesions. Interestingly, the proportion of DCIS lesions with positive p53 staining actually decreased in DCIS Subgroup II*a*, which had the same molecular subtypes as both the adjacent TDLU and IDC (*P* = 0.02). In contrast, we found that DCIS lesions in Subgroup II*b*, which had the same molecular subtypes as the adjacent IDC but not the same subtypes as the adjacent TDLU, had a higher Ki-67 index (*P*<10^−7^) and a higher likelihood of positive p53 staining (*P* = 0.02), and less p16 staining (*P* = 0.08, borderline) than those in type I DCIS lesions with different molecular subtypes from the adjacent IDC. We also considered logistic regression models involving Ki67, p53 and p16 with the goal of identifying the models(s) that best predicted sub-group II*b*. A gradual improvement in AUC was observed with the inclusion of Ki67, either alone or in conjunction with p53 and/or p16, with the best performing model being that containing all markers [AUC = 0.82, 95% CI: (0.78,0.86)].

**Table 4:** Association between IHC marker scores and individual DCIS lesions classified based on molecular subtypes of adjacent IDC and TDLU from patients with IDC and DCIS (n = 36).

DCIS Classification^a^		Ki-67			p16			p53	
Subgroups	Subtypes vs. IDC or TDLU	Low^b^	Moderate (*P*)^c^	High (*P*)	High^b^	Moderate (*P*)	Low (*P*)	Wild-type^b^	Mutant (*P*)
I	Not same as IDC^b^	79	42	4	18	54	51	119	7
II*a*	Same as IDC and TDLU	50	46 (*0.24*)	2 (*0.69*)	15	25 (*0.28*)	54 (*0.84*)	99	0 (***0.02***)
II*b*	Same as IDC but not as TDLU	14	56 (***2.1×10^−8^***)	23 (***1.5×10^−10^***)	8	30 (*0.64*)	53 (*0.08*)	76	15 (***0.02***)

a DCIS lesions from each case were first classified into two subgroups based on the molecular subtypes of adjacent IDC: Subgroup I DCIS, which presented different molecular subtypes from the adjacent IDC; Subgroup II DCIS, which presented the same molecular subtypes as the adjacent IDC. Then the Subgroup II DCIS lesions were further divided into Subgroups II*a* and II*b* based on the molecular subtypes of adjacent TDLU.

b Reference category.

c P-value (Fisher's exact test).

## Discussion

**Figure 3:**
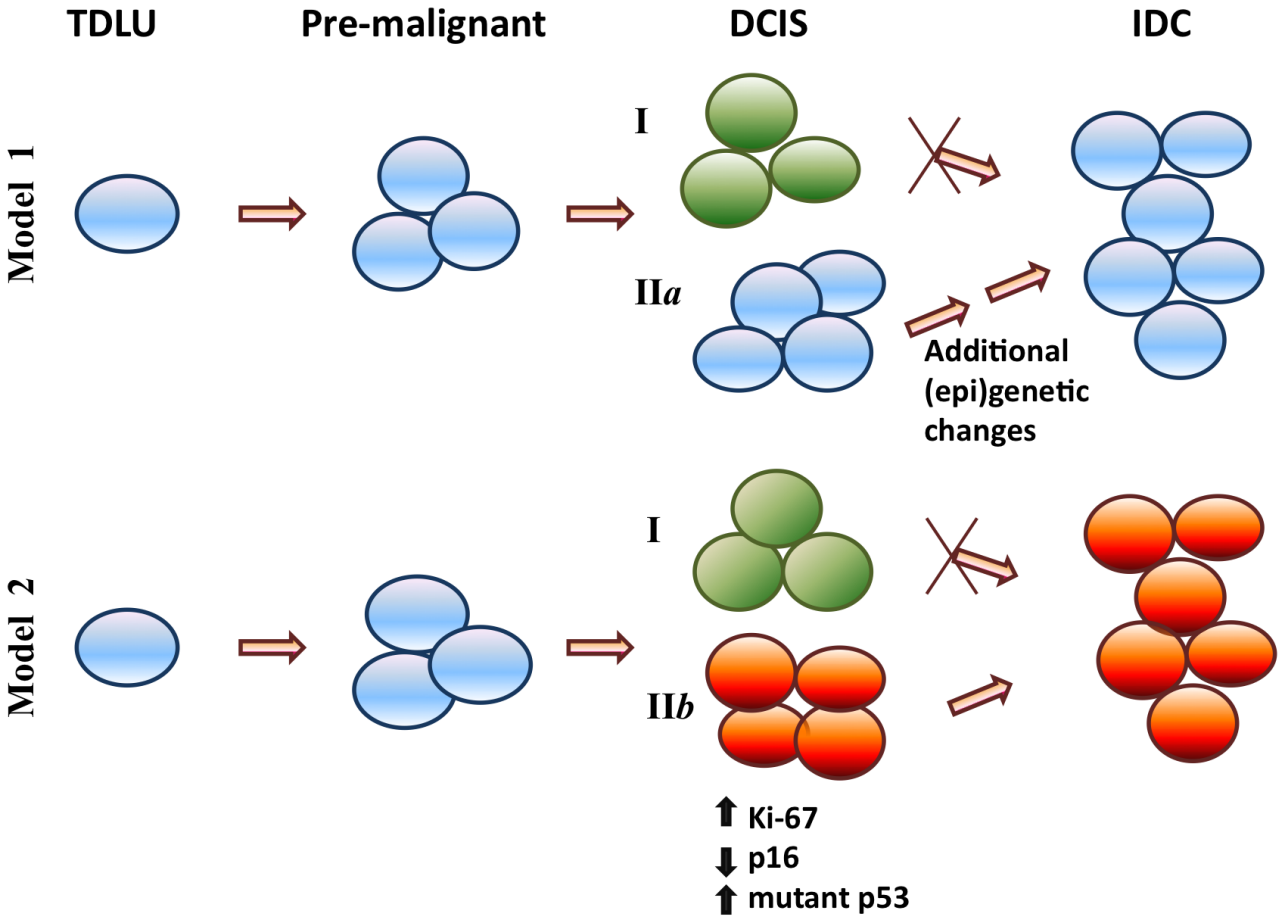
Identification of a “highly-aggressive” DCIS subgroup. DCIS lesions are sub-classified according to the molecular subtypes of adjacent TDLU and IDC (**Model 1**: TDLU and IDC have the same molecular subtypes; **Model 2**: TDLU and IDC have different molecular subtypes). By comparing Ki-67, p53 and p16 IHC scores, DCIS Subgroup II*b*, which had the same molecular subtypes as the adjacent IDC but not the same subtypes as the adjacent TDLU, represents a “highly-aggressive” or “high-risk” subpopulation of DCIS which are putatively direct precursor of adjacent IDC, while DCIS Subgroups I and likely II*a* represent “less-aggressive” or “low-risk” subpopulations which either require additional (epi)genetic changes or are not able to directly progress into IDC.

Heterogeneity of invasive ductal carcinoma cells both within and between individuals at the morphological, molecular and immunohistochemical levels has been documented extensively [33], [34]. Here, we add another level of complexity to the heterogeneous nature of breast cancer precursors by reporting that multiple DCIS lesions from the same patient with IDC frequently exhibit heterogeneity in the expression of clinically relevant markers. Six commonly used IHC markers were investigated in the present study. For the first time, intra-individual heterogeneity was measured quantitatively by taking into account both the variability in the expression of individual markers and the frequencies of the markers harboring expression variability for each case. PR, HER2, Ki-67, and p16, but not ER and p53, displayed significantly diverse expression patterns in DCIS lesions within an individual patient, ranging from 20% to 60% of the cases (**Figure 1B**). In addition, seventy-two percent of the individuals had heterogeneous expression in at least 2/6 markers, which significantly differed from our hypothesized homogeneous baseline of 10% of cases demonstrating heterogeneity (*P*<10^−18^) (**Figure 1C**). These findings confirmed that intra-individual heterogeneity in multi-lesional DCIS is quite common. We also demonstrated that 38.9% cases (14 out 36) had DCIS lesions with variable molecular subtypes (**Figure 2B**). Not surprisingly, we found that HER2+ and basal-like subtype DCIS lesions have higher Ki-67 and p53 scores than DCIS lesions with the luminal A subtype (**Table 3**). DCIS lesions with different molecular subtypes were then categorized into distinct groups by comparing their subtypes with those of adjacent normal TDLU and IDC (**Figure 2C**). Importantly, by comparing the expression of promising DCIS risk biomarkers (Ki-67, p53 and p16) among different DCIS subgroups, our results suggest the existence of a highly-aggressive DCIS subgroup, which had the same molecular subtype as the adjacent IDC but not the same subtype as the adjacent normal terminal duct lobular units (TDLU) (**Table 4**). Prior reports suggested that high Ki-67 index, positive p53 expression, and loss of p16 represent typical characteristics for invasive breast cancer and are promising biomarkers for DCIS progression [35]–[38]. Therefore, our results suggest that DCIS Subgroup II*b* obtains these “aggressive” characteristics, thus they represent a “high-risk” subpopulation of DCIS lesions which could be the direct precursors to the adjacent IDC. DCIS Subgroups I and likely II*a* represent a “less-aggressive” or “low-risk” subpopulation which may not progress directly into IDC without additional (epi)genetic changes (**Figure 3**). Furthermore, lesions from DCIS subgroup II*b* had a higher tumor grade than those from subgroup I (*P* = 0.002) (**Data not shown**); this result further supports that DCIS subgroup II*b* may represent an “aggressive” type of DCIS.

Consistent with the breast cancer progression model of Welling and Jensen [9], [10], results from the present study suggest that some (epi)genetic changes must arise in an early precursor cell and are inherited by daughter cells that become increasingly abnormal as they mature (i.e. DCIS). In order for the cells to progress to IDC, DCIS cells must acquire additional genetic or epigenetic alterations that confer a survival advantage for these cells over others without the alterations (DCIS that does not progress). This breast tumor progression model may explain why some DCIS patients, but not others, subsequently develop IDC. Most likely, not every DCIS lesion within the same patient has the ability to progress to IDC because of the intra-individual heterogeneity among DCIS lesions. Only those lesions with more aggressive characteristics, including but not limited to, high proliferation rate (high Ki-67), gain of mutant p53, and loss of the tumor suppressor p16, are favorable for breast cancer progression. Interestingly, our data showed that a high-degree of heterogeneity in DCIS lesions may also be associated with positive nodal involvement in comparison to the homogeneous group (*P* = 0.07, borderline, **Table 2**). This data therefore suggests that those individuals with a heterogeneous DCIS cell population combined with high levels of Ki-67, increased mutant p53, and low p16 should be clinically managed more aggressively. However, our findings are based on a small number of clinical samples from patients concurrently diagnosed with IDC and DCIS. Future validation studies with a large sample set are warranted.

To overcome the challenges caused by intra-individual heterogeneity of DCIS, several strategies should be considered when establishing new DCIS biomarkers. First, not only should a reasonably large number of samples be evaluated to achieve statistical significance, but multiple biopsies from the same patient should also be evaluated to avoid the bias caused by intra-individual heterogeneity. Secondly, it is imperative when developing biomarkers of DCIS progression or chemotherapeutic response, to use adjacent normal and invasive tumor cells whenever possible from the same individual as controls. This approach will help to decrease the variability in the results from multiple individuals. Finally, new alternative research strategies that take advantage of the intra-individual heterogeneity in DCIS should be explored as described in this study. Our results support a new approach in which a case (i.e. high-risk DCIS Subgroup II*b*) *vs*. control (i.e. low-risk DCIS Subgroup I) cohort could be used to facilitate the discovery of DCIS risk markers. We believe that the new approach proposed here could hasten biomarker discovery by overcoming the challenges (e.g., evident inter-individual heterogeneity in DCIS patients) faced in conventional comparison between “pure” DCIS and DCIS with IDC.

In summary, this study is the first quantitative assessment to reveal the common presence of intra-individual heterogeneity in the expression of several selected markers in DCIS lesions from patients concurrently diagnosed with IDC. The heterogeneous nature of DCIS lesions within the same individual speaks clearly to the need to develop a better strategy to study the progression of DCIS. Here we established a novel approach to evaluate heterogeneity in DCIS by comparing the molecular subtypes of adjacent TDLU and IDC with DCIS lesions. Our new approach ultimately leads to the discovery of a distinct “aggressive” subgroup of DCIS lesions. As the likely precursors of adjacent IDC, studying the heterogeneity of DCIS within the same patient could provide new insight into the identification of risk factors for DCIS progression as well as personalized risk assessment for this breast cancer precursor. Since heterogeneous pre-invasive lesions are also present in other cancer types (e.g. prostate cancer, colon cancer), our findings about the sub-classification of *in situ* carcinoma in the patients concurrently diagnosed with invasive disease could have broad implications for studying tumor progression in many cancer types.

## Supporting Information

File S1 **Supporting information file contains Table S1, Table S2, Figure S1, and Figure S2.** **Table S1**. Associations between IHC marker scores among individual DCIS lesions from patients with IDC and DCIS (n = 36). **Table S2**. Associations between Ki-67 and Cleaved Caspase-3 scores among individual DCIS lesions from patients with IDC and DCIS (n = 36). **Figure S1. Immunohistochemical scoring guidelines for ER, PR, HER-2, p16, Ki-67, p53, p63 and Cleaved Caspase-3.** H-scores that were below 50 were considered negative for ER and PR, whereas H-scores above 50 were considered positive for ER and PR. Membranous expression of HER2 in DCIS lesions was scored on a scale of 0–3. Ki-67 and p53 scoring was based on the percentage of cells with strongly stained nuclei. For p53 staining only lesions containing cells with very strongly stained nuclei. H-scores for p16 were determined by multiplying the intensity of stained cells (0, 1, 2, 3) by the percentage of positive cells. Staining of Cleaved Caspase-3 was then classified in two categories: negative-to-low (if H-score <2) or medium-to-high (if H-score ≥2). IHC staining of p63 was performed to ensure that the ductal carcinoma cells were still bound by a myoepithelial cell layer. **Figure S2. Kaplan-Meier survival curves for overall survival.** (**A**) and recurrence-free survival (**B**) in patients with IDC and DCIS (n = 36).
(DOCX)
