# Supplementary material for: Identifying a Highly-Aggressive DCIS Subgroup by Studying Intra-Individual DCIS Heterogeneity among Invasive Breast Cancer Patients
Source: PLoS One. 2014 Jun 30;9(6):e100488. doi: 10.1371/journal.pone.0100488 (PMC4076240; doi:10.1371/journal.pone.0100488)

**Table S1.** Associations between IHC marker scores among individual DCIS lesions from patients with IDC and DCIS (n=36).

| **Scores of IHC Markers** | **ER Positive (%)** | **ER Negative (%)** | **O.R.** (95% CI) | ***P*-Value** ^b^ |
| --- | --- | --- | --- | --- |
| PR Positive ^a^  PR Negative | 174 (73.1%) | 12 (14.0%) |  |  |
|  | 64 (26.9%) | 74 (86.0%) | 16.8 (8.5, 32.9) | ***3.3 × 10 ^-22^*** |
| HER2 Low (Score 0-1) ^a^  HER2 High (Score 2-3) | 39 (16.5%) | 18 (20.7%) |  |  |
|  | 198 (83.5%) | 69 (79.3%) | 0.76 (0.48, 1.31) | *0.41* |
| Ki-67-Low (Score 1) ^a^  Ki-67-Med (Score 2)  Ki-67-High (Score 3) | 141 (58.0%) | 14 (15.7%) |  |  |
|  | 88 (36.2%) | 58 (65.2%) | 6.64 (3.50, 12.6) | ***2.3 × 10 ^-10^*** |
|  | 14 (5.8%) | 17 (19.1%) | 12.2 (4.99, 30.0) | ***4.3 × 10 ^-8^*** |
| p16-High (Score 3) ^a^  p16-Med (Score 2)  p16-Low (Score 1) | 30 (12.8%) | 11 (12.6%) |  |  |
|  | 73 (31.0%) | 45 (51.8%) | 1.68 (0.77, 3.68) | *0.25* |
|  | 132 (56.2%) | 31 (35.6%) | 0.64 (0.29, 1.42) | *0.28* |
| p53 Wild-type (Score 0) ^a^  p53 Mutant (Score 1-3) | 238 (97.9%) | 69 (78.4%) |  |  |
|  | 5 (2.1%) | 19 (21.6%) | 13.1 (4.72, 36.4) | ***3.2 × 10 ^-8^*** |

*^a^ Reference category.*

*^b^ Fisher’s exact test.*

**Table S2.** Associations between Ki-67 and Cleaved Caspase-3 scores among individual DCIS lesions from patients with IDC and DCIS (n=36).

| **Cleaved Caspase-3** | **Ki-67** | | |
| --- | --- | --- | --- |
|  | Low ^a^ | Moderate (*P* ^b^) | High (*P*) |
| Negative to low (H-score < 2) ^a^ | 125 | 69 | 11 |
| Medium to high (H-score ≥ 2) | 26 | 70 (***2.5 × 10 ^-9^***) | 20 (***3.3 × 10 ^-7^***) |

^a^ Reference category.

^b^ *P-value (Fisher’s exact test).*

**Figure S1.**

**
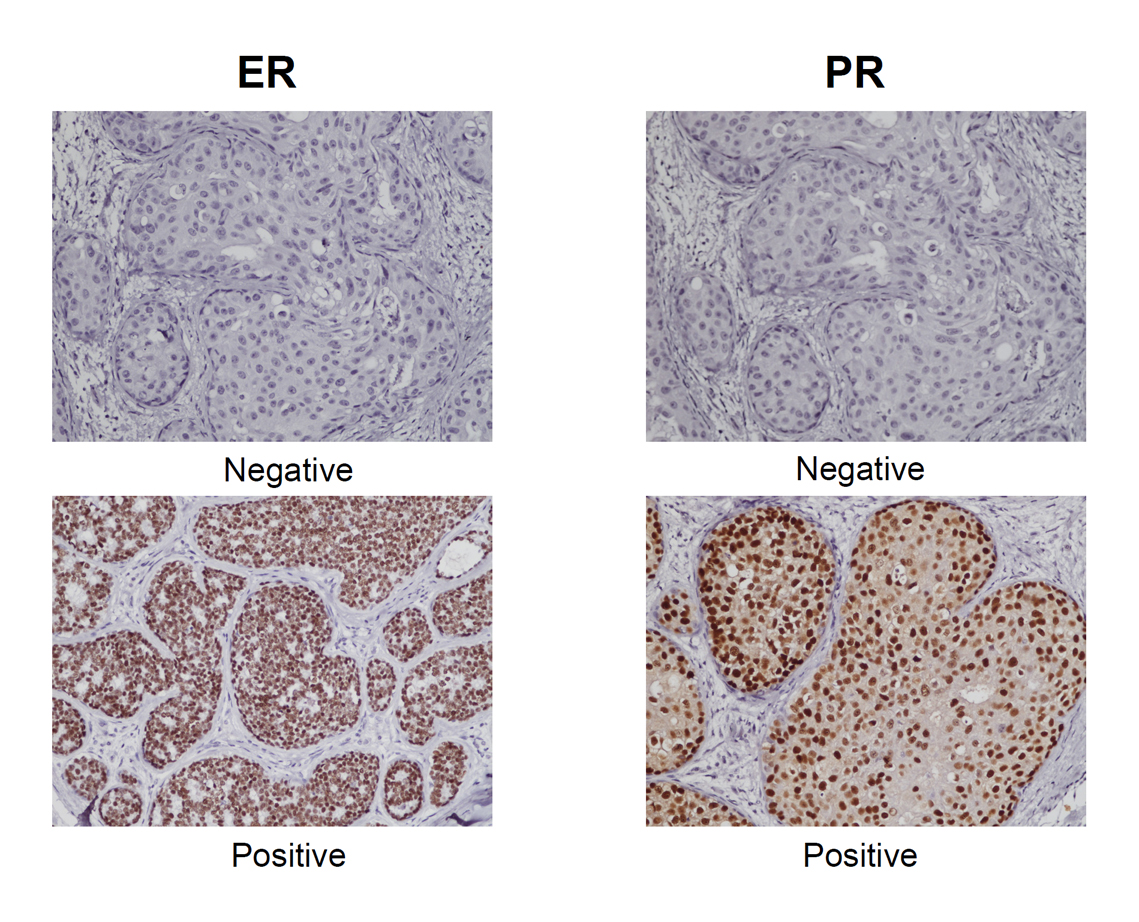
**

**
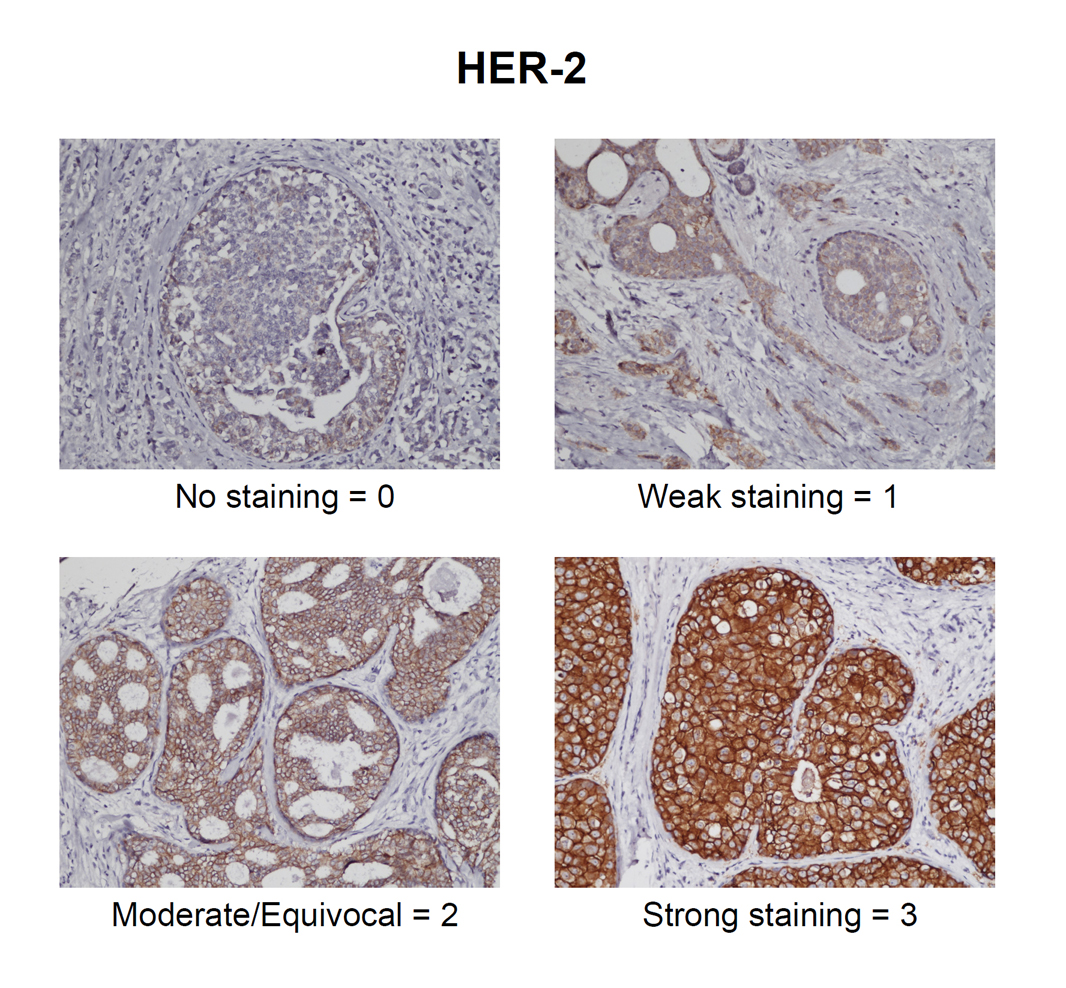
**

**
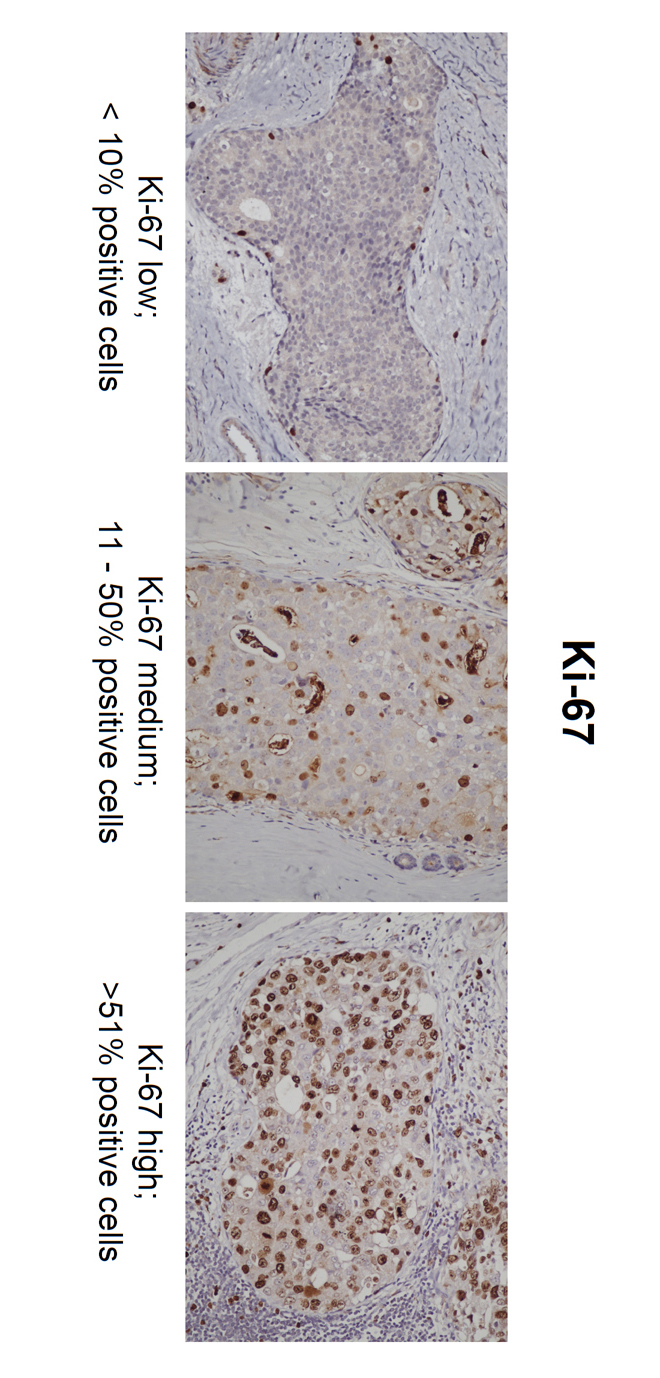
**


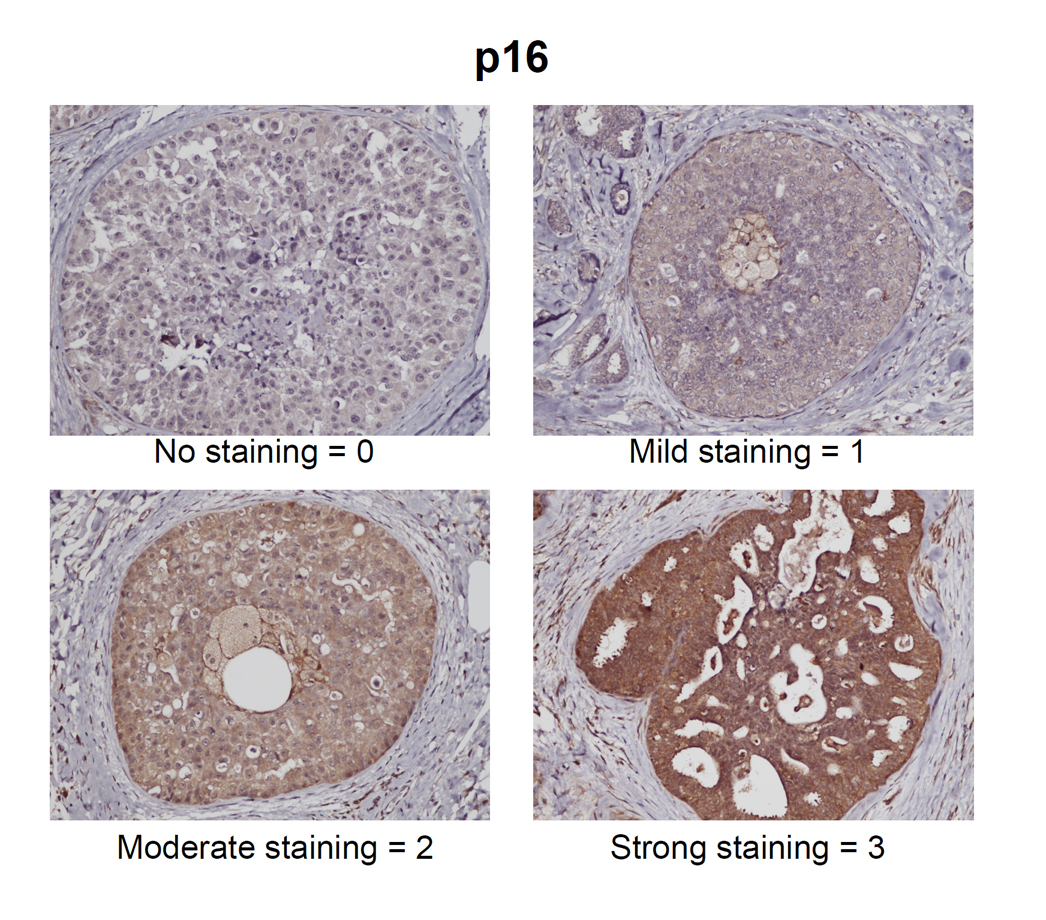


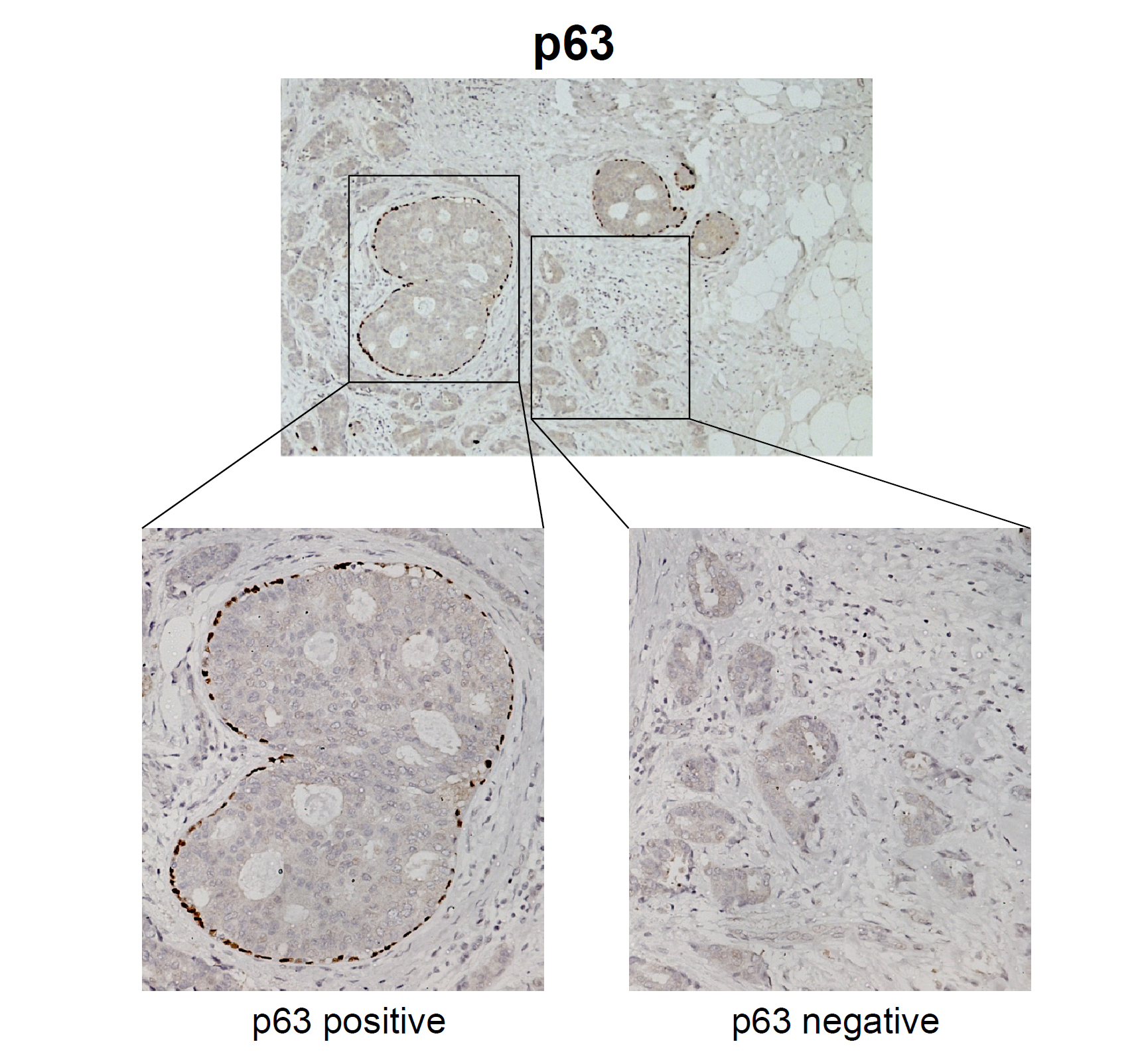


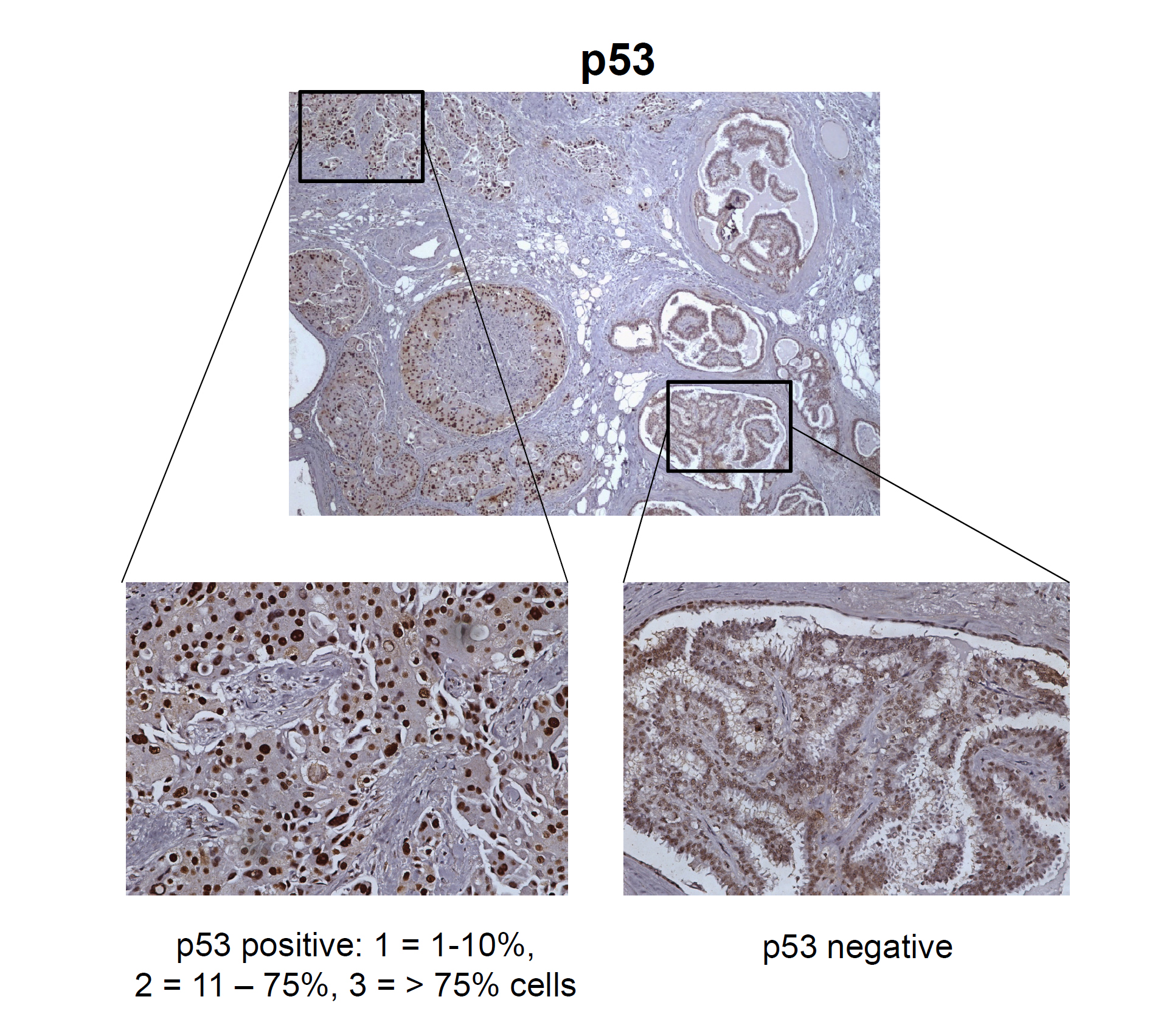


**
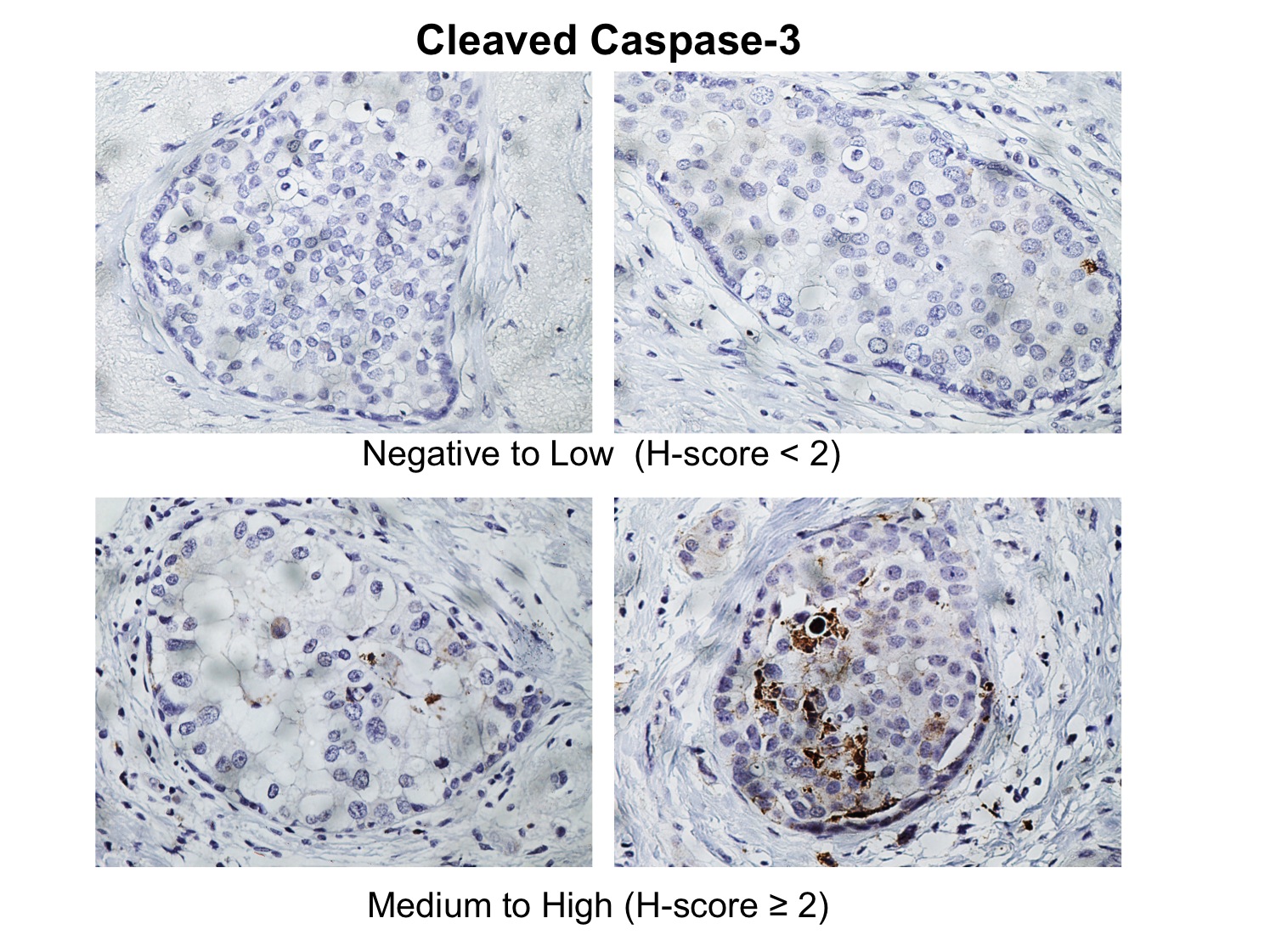
**

**Figure S2.**

**A. B.**

**
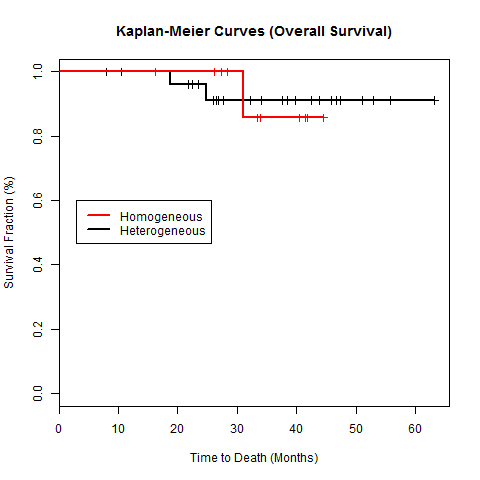
**


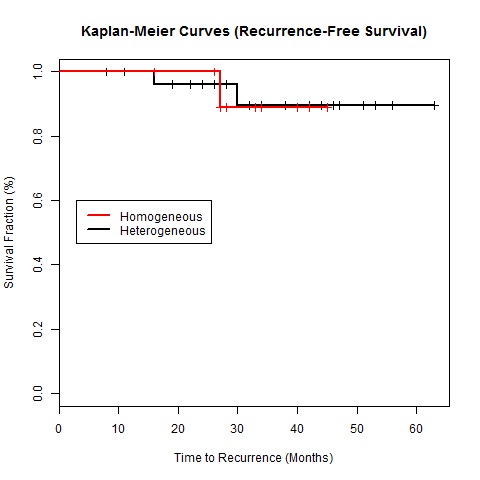

Supplement: File S1 — Supporting information file contains Table S1, Table S2, Figure S1, and Figure S2. Table S1. Associations between IHC marker scores among individual DCIS lesions from patients with IDC and DCIS (n = 36). Table S2. Associations between Ki-67 and Cleaved Caspase-3 scores among individual DCIS lesions from patients with IDC and DCIS (n = 36). Figure S1. Immunohistochemical scoring guidelines for ER, PR, HER-2, p16, Ki-67, p53, p63 and Cleaved Caspase-3. H-scores that were below 50 were considered negative for ER and PR, whereas H-scores above 50 were considered positive for ER and PR. Membranous expression of HER2 in DCIS lesions was scored on a scale of 0–3. Ki-67 and p53 scoring was based on the percentage of cells with strongly stained nuclei. For p53 staining only lesions containing cells with very strongly stained nuclei. H-scores for p16 were determined by multiplying the intensity of stained cells (0, 1, 2, 3) by the percentage of positive cells. Staining of Cleaved Caspase-3 was then classified in two categories: negative-to-low (if H-score <2) or medium-to-high (if H-score ≥2). IHC staining of p63 was performed to ensure that the ductal carcinoma cells were still bound by a myoepithelial cell layer. Figure S2. Kaplan-Meier survival curves for overall survival. (A) and recurrence-free survival (B) in patients with IDC and DCIS (n = 36). (DOCX) [file pone.0100488.s001.docx]
